# Complex Challenges in ANCA-Associated Vasculitis: A Case of COVID-19, Cytomegalovirus Pneumonitis, and Hemorrhagic Cholecystitis Following Rituximab Therapy

**DOI:** 10.7759/cureus.108480

**Published:** 2026-05-08

**Authors:** Fatema Ezzy, Dina Ismail, Tiago Reyes Castro, Chen Chao, Nana Jinjolava

**Affiliations:** 1 Internal Medicine, Jacobi Medical Center, Albert Einstein College of Medicine, Bronx, USA; 2 Family Medicine, Faculty of Medicine and Pharmacy, University Hassan II of Casablanca, Casablanca, MAR; 3 Internal Medicine, New York Medical College, Metropolitan Hospital Center, New York, USA; 4 Rheumatology, Montefiore Medical Center, Albert Einstein University, Bronx, USA; 5 Rheumatology, Jacobi Medical Center, Albert Einstein College of Medicine, Bronx, USA

**Keywords:** antineutrophil cytoplasmic antibody (anca) associated vasculitis (aav), congenital cytomegalovirus infection, covid-19, hemorrhagic cholecystitis, immune reconstitution inflammatory syndrome, rituximab therapy

## Abstract

Antineutrophil cytoplasmic antibody (ANCA)-associated vasculitis (AAV) is a rare autoimmune disease with significant infectious and hemorrhagic risks during immunosuppressive therapy. We describe an 81-year-old woman with perinuclear ANCA (p-ANCA)-associated vasculitis complicated by acute respiratory distress syndrome from concurrent COVID-19 and CMV pneumonitis following rituximab induction. Her course was further complicated by hemorrhagic cholecystitis, a rare bleeding manifestation, requiring percutaneous intervention. Careful adjustment of immunosuppression was necessary due to severe infections. This case highlights the complexity of managing AAV amid severe infections and rare hemorrhagic complications, emphasizing the need for vigilant monitoring and individualized therapy.

## Introduction

Antineutrophil cytoplasmic antibody (ANCA)-associated vasculitis (AAV) is a rare autoimmune condition primarily affecting small blood vessels and various organ systems, including the kidneys, respiratory system, and skin. Before the advent of immunosuppressive therapies, AAV exhibited a mortality rate of 93% within two years, primarily attributable to renal and respiratory failure [1,2]. The introduction of induction agents such as glucocorticoids in 1948, cyclophosphamide in the 1960s, and, most recently, rituximab in 2010 has significantly improved survival outcomes, with current five-year survival rates approaching 80% [3-5]. Nonetheless, AAV is still associated with a seven-fold increase in the risk of death compared to the general population [6]. The majority of deaths within the initial year of diagnosis now result from therapy-associated adverse events (59%) rather than active vasculitis (14%). Although the incidence varies widely, studies have estimated that infections account for 38.8%-85% of these therapy-associated deaths [3,7,8]. Despite this, the rarity of AAV makes it difficult to gather high-quality data to guide immunosuppressive strategies while managing these infectious complications, especially during the induction phase of therapy.

We present a unique case of an 81-year-old woman diagnosed with AAV, whose clinical course was complicated by acute respiratory distress syndrome secondary to COVID-19, hemorrhagic cholecystitis, and cytomegalovirus (CMV) infection after receiving induction therapy with rituximab and high-dose steroids. Here, we aim to outline the management of immunosuppressive therapy in light of these complications, given the lack of current evidence to guide clinicians in managing severe infectious complications related to AAV treatment.

## Case presentation

**Figure 1:**
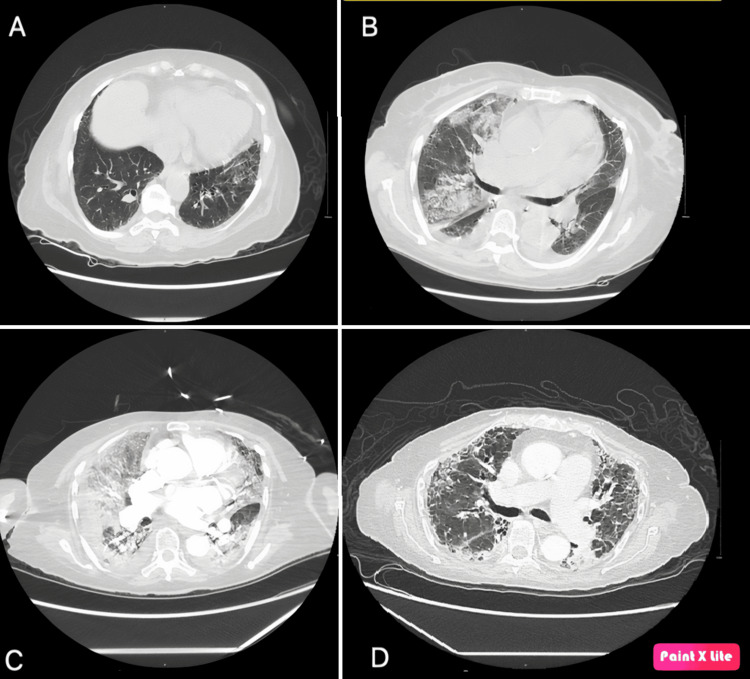
Imaging timeline of interstitial lung disease complicated by COVID-19 pneumonia and subsequent recovery. (A) Interstitial lung disease identified at the time of diagnosis. (B) Development of new patchy opacities and small bilateral pleural effusions with COVID-19 pneumonia. (C) Interval worsening of diffuse ground-glass opacities with more confluent areas of consolidation superimposed on interstitial lung disease. (D) Follow-up chest CT three months later with interstitial lung disease and interval resolution of diffuse ground-glass opacities and consolidation.

An elderly woman in her 80s presented to the clinic one week after receiving induction therapy for newly diagnosed microscopic polyangiitis (a subtype of AAV). She reported blood-streaked sputum with respiratory distress following symptoms of an upper respiratory tract infection. She had recently been diagnosed with glomerulonephritis and interstitial lung disease secondary to AAV three weeks ago (Figure 1), and treatment had been initiated with a pulse dose of steroids and intravenous rituximab [4]. Cardiac workup at the time showed mild pulmonary hypertension with a normal ejection fraction (58%) and no significant structural or valvular abnormalities. Her home medications included oral prednisone dosed at 60 mg along with *Pneumocystis jirovecii* prophylaxis with atovaquone. Her medical history was significant for hypertension and prediabetes. She had no other heart diseases, had never smoked, and was fully vaccinated, having received all three doses of the COVID-19 mRNA vaccines. Of note, she had recently traveled to the Dominican Republic 3 months ago.

In the office, her vital signs were stable, but the physical examination was significant for diffuse inspiratory crackles and bilateral pitting edema. Given the complexity of the differential diagnoses in an immunosuppressed patient with non-massive hemoptysis, she was sent to the emergency department for a comprehensive evaluation.

In the hospital, she progressively developed acute hypoxic respiratory failure, requiring oxygen support with a nasal cannula. Her laboratory values were remarkable for a normal leukocyte count but a high neutrophil-to-lymphocyte ratio (50), indicating severe disease [9] and elevated inflammatory markers (Table 1). SARS-CoV-2 was detected via reverse transcriptase polymerase chain reaction (RT-PCR) test with a cycle threshold (ct) value of 20.9, and computed tomography (CT) of the chest showed new right-sided, ground-glass and patchy opacities without evidence of pulmonary embolism (Figure 1).

**Table 1:** Laboratory trends before admission, on admission, and during hospitalization in an 81-year-old patient with ANCA-associated vasculitis. BAL: bronchoalveolar lavage; N/A: not available; PCR: polymerase chain reaction; proBNP: pro-B-type natriuretic peptide; RBC: red blood cell; SARS CoV-2: severe acute respiratory syndrome coronavirus-2; SGPT: serum glutamic pyruvic transaminase; SGOT: serum glutamic oxalo-acetic transaminase; WBC: white blood cell; nL: nanoliters; g/dL: grams per deciliter; mEq/L: milliequivalents per liter.

Laboratory component	Results before admission	Results on admission	Results 12 days after admission	Reference range
Leukocytes (WBC)	6.41	10.81	10	3.5-11 /nL
Absolute lymphocyte count	0.18	0.21	0.5	1.2-3.5 /nL
Hemoglobin	7.5	8	8.9	12-16 g/dL
Platelets	195	194	91	150-400 /nL
Sodium	134	135	144	135-145 mEq/L
Potassium	4.6	4.6	4.4	3.5-5 mEq/L
Creatinine	3.1	2.7	2.2	0.5-1.5 mg/dL
Blood urea nitrogen	62	54	47	5-26 mg/dL
SGPT	17	21	N/A	1-40 U/L
SGOT	12	15	N/A	1-40 U/L
Alkaline phosphatase	59	81	N/A	35-104 U/L
Total bilirubin	0.3	0.4	N/A	0.1-1.2 mg/dL
Total albumin	3	3.4	N/A	3.5-5.5 g/dL
Total protein	5	5.5	N/A	6-8.5 g/dL
Prothrombin time	11.8	11.8	12.3	9.4-12.5 seconds
Activated thromboplastin time	26	27.9	N/A	25.1-36.5 seconds
C-reactive protein	132.5	18.8	21.9	0-5 mg/dL
Erythrocyte sedimentation rate	32	23	N/A	0-30 mm/h
Troponin	N/A	N/A	0.050	0.0-0.090 ug/L
ProBNP	N/A	N/A	20,421	1-450 pg/nL
SARS CoV-2 PCR	N/A	Positive	Positive	Negative
SARS CoV-2 cyclic threshold	N/A	20.9	34	1-45 cycles
Procalcitonin	N/A	0.11	0.35	0.02-0.08 ng/mL
Fungitell B-D Glucan	N/A	<31	36	<60 pg/mL
Myeloperoxidase Antibody	135.8	81.5	44.3	<20 units
BAL total nuclear cells including WBCs	N/A	391	273	/uL
BAL RBCs	N/A	14,000	10,000	/uL
BAL polynuclear cells	N/A	84.9	36.6	%
BAL mononuclear cells	N/A	15.5	63.4	%
BAL neutrophils	N/A	85	32	%
BAL lymphocytes	N/A	5	20	%
BAL monocytes	N/A	10	48	%

In light of these investigations, treatment was initiated for COVID-19 pneumonia with intravenous remdesivir and dexamethasone [10]. Concurrently, broad-spectrum antibiotics and trimethoprim-sulfamethoxazole were started due to concern for superimposed pneumonia with bacterial and opportunistic agents like pneumocystis jirovecii, given recent immunosuppression despite adequate prophylaxis with atovaquone. Inhaled tranexamic acid for symptomatic management of hemoptysis was also started.

Overnight, she developed worsening acute hypoxic respiratory failure with septic shock, requiring intubation with mechanical ventilatory support, proning, and vasopressor support. Chest imaging showed worsening bilateral opacities (Figure 1). We broadened our differentials to septic shock, acute respiratory distress syndrome (ARDS), and diffuse alveolar hemorrhage (DAH). Bronchoscopy was pursued, and serial lavages ruled out DAH. Intravenous tocilizumab (8 mg/kg) was administered for severe COVID-19 pneumonia [11]. In the following days, she improved clinically, with successful extubation nine days later. Her decreasing oxygen requirements were fulfilled by a nasal cannula. As extensive blood and bronchoalveolar lavage tests and cultures were negative for an alternative causative agent, including bacteria, fungi, parasites, and other viruses, treatment was tailored for severe COVID-19 pneumonia with ARDS. Antibiotics and antifungals were discontinued. The only immunosuppression she received during this period was the dexamethasone dosed for COVID-19 pneumonia (equivalent to 40 mg of oral prednisone).

On the 12th day, there was a sudden deterioration in her respiratory status, necessitating re-intubation and mechanical ventilatory support. Physical examination revealed tachypnea and bilateral rales on auscultation. Laboratory values were notable for a normal leukocyte count with an NLR indicating moderate disease (17.8), thrombocytopenia (91/nL), low procalcitonin level (0.35), elevated but improved creatinine and blood urea nitrogen (BUN) levels, and elevated pro-B-type natriuretic peptide (proBNP) levels (20,421 pg/mL) (Table 1). 

Our differential diagnoses included delayed clearance of COVID-19 infection, superimposed bacterial infection, opportunistic fungal, viral, or parasitic infections (given steroid use and recent travel history), DAH, AAV flare triggered by COVID-19 infection, new onset heart failure, and pulmonary embolism. 

Further workup showed a negative respiratory viral panel except for SARS-CoV-2 with an increasing ct value of 34, depicting clearance of the viral infection, albeit slow. Imaging ruled out pulmonary embolism but noted worsening bilateral and diffuse pulmonary opacities. A transthoracic echocardiogram showed mildly increased right-sided pressures with preserved ejection fraction. This could be attributable to the severe lung disease in the absence of other factors such as pulmonary embolism. Bronchoscopy was repeated, which showed thick secretions without evidence of DAH. Bronchial cultures did not show any bacterial, fungal, or parasitic growth. This reduced the likelihood of many infectious agents as potential causes. High CMV titers (192,000 IU/mL) were detected in the serum through PCR. Unfortunately, viral cultures from bronchoalveolar lavage could not be sent due to technical processing issues, and she remained hemodynamically unstable to undergo further confirmation of tissue invasive disease. Our narrowed list of potential diagnoses included delayed COVID-19 infection, the possibility of CMV pneumonia, and the coexistence of an AAV flare concurrent with right heart failure.

Treatment involved a broad therapeutic approach encompassing induction therapy with intravenous ganciclovir (85 mg daily for two weeks), followed by oral valganciclovir therapy until negative CMV titers were detected for possible CMV pneumonia (based on definitions outlined by the CMV Drug Development Forum [12]). Following a collaborative discussion involving the critical care team and rheumatology, a brief course of steroid mini-pulse therapy was initiated. This involved administering intravenous methylprednisolone at a dosage of 125 mg daily for three days, given that vasculitis flare was still in the differentials. Concurrently, one dose of convalescent plasma was administered for possible COVID-19 infection. Antifungals and antibiotics were initially prescribed empirically but later discontinued.

**Figure 2:**
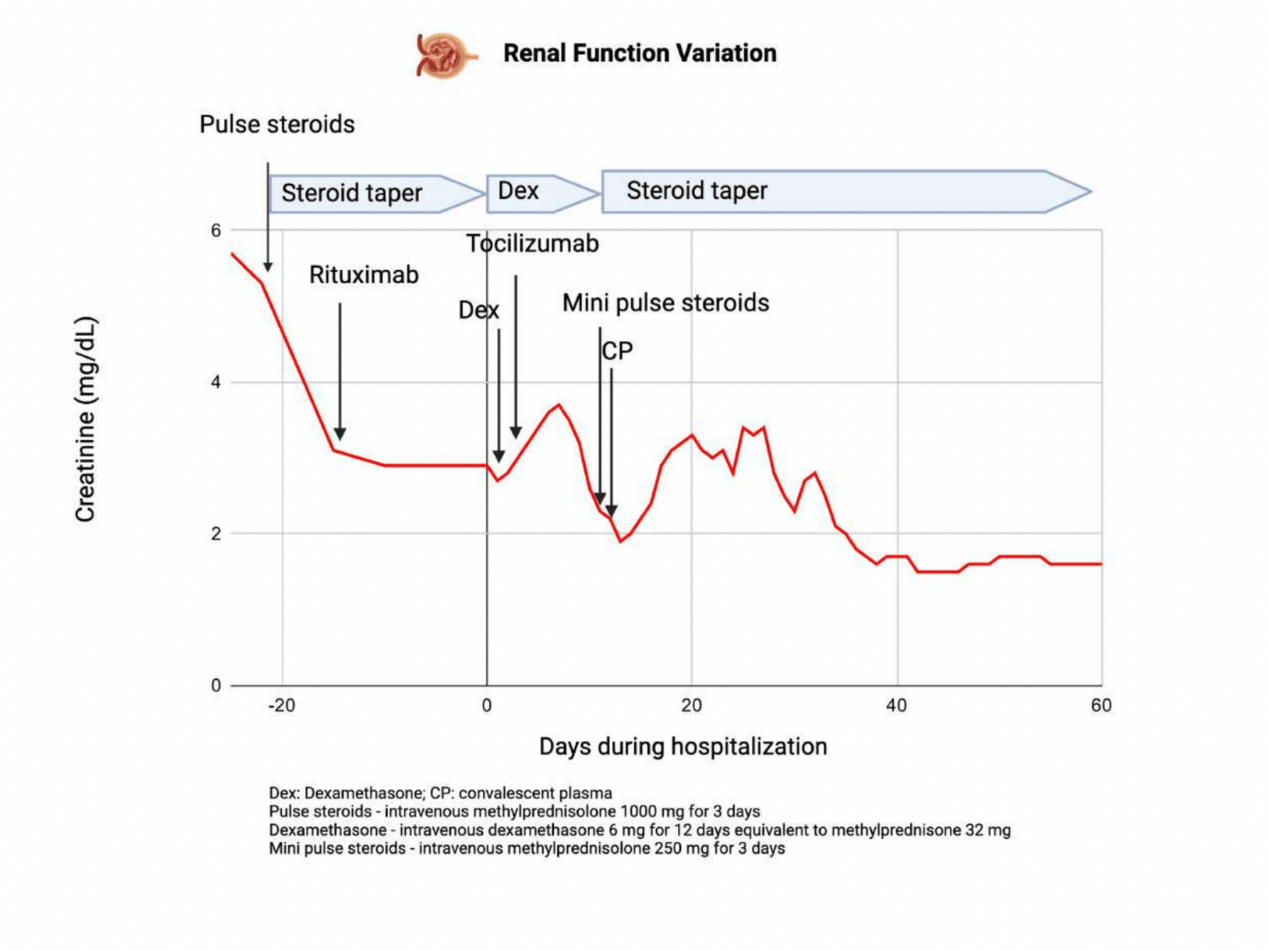
The variation in renal function over the course of the hospitalization. During the time AAV was diagnosed, her creatinine levels were 5.3 mg/dL, which had not been responsive to hydration. Rapid renal improvement was seen after administering a pulse dose of steroids as part of the induction therapy. Rituximab was initiated along with steroid taper, initially at 60 mg of oral prednisone. Upon hospital admission, immunosuppression was modified to include dexamethasone (equivalent to 40 mg of oral prednisone), and rituximab was temporarily discontinued. Subsequently, due to ongoing treatment for infectious processes, steroids were increased to the equivalent of 60 mg of oral prednisone following mini-pulse administration, while rituximab continued to be held. Concurrently, her renal function deteriorated transiently, attributable to various factors, including septic shock, contrast exposure during CT imaging, fluctuating volume status, and urinary retention. Remarkably, her renal function stabilized at a creatinine level of 1.6 mg/dL towards the end of her hospital course. AAV: ANCA-associated vasculitis. Image created with BioRender.com (BioRender.com Inc., Toronto, ON, Canada).

This regimen resulted in clinical improvement and successful extubation two days later. She continued to improve with oxygen requirements reducing to using a nasal cannula. Her renal function also improved concurrently (Figure 2). Steroid taper with valganciclovir therapy was continued. The second dose of rituximab was delayed in anticipation of completion of maintenance therapy with valganciclovir. She was eventually discharged to a subacute rehabilitation center to continue physical therapy.

After consulting with the patient, her immunosuppression was transitioned to a daily dose of 50 mg of oral azathioprine while maintaining the steroid taper. A subsequent influenza A infection led to her readmission to the hospital within two months. Unfortunately, azathioprine had to be discontinued due to the emergence of liver injury and severe leukopenia while being treated for influenza A infection, attributable to the drug. The primary focus of her AAV treatment continued to be the steroid taper, with which she was discharged. The challenge of further immunosuppression persisted. Encouragingly, her creatinine levels exhibited improvement, decreasing to 1.2 mg/dL during the three-month follow-up (Figure 2). Additionally, a chest CT scan revealed the resolution of bilateral opacities (Figure 1). 

## Discussion

In recent years, the impact of COVID-19 on patients with AAV has been notable for contributing to their increased morbidity and mortality. Interestingly, the incidence of COVID-19 in individuals with AAV aligns closely with that of the general population [13]. Various risk factors have been identified as contributing to poorer outcomes in these patients, including older age, a glucocorticoid dosage of 10 mg/day or higher, moderate to severe disease activity, a high number of comorbidities, and the use of rituximab or cyclophosphamide [14]. The role of immunosuppressive agents is particularly noteworthy in this context. In addition to increasing severity and mortality, administering glucocorticoids at a daily dose exceeding 10 mg prednisone (or equivalent) is associated with an increased likelihood of contracting COVID-19 and subsequently delaying the viral clearance [15]. 

Given the presence of several risk factors associated with poor outcomes in our patient, we reviewed the current literature for guidance on her management. In randomized controlled trials examining the effects of rituximab versus cyclophosphamide and plasma exchange versus glucocorticoids in AAV, a swift prednisone taper for remission induction was favored due to diminished benefits and heightened infection risks at higher steroid doses [8,16]. Additionally, a prednisone equivalent of 40 mg has shown improved 28-day mortality in severe COVID-19 cases requiring oxygen support [2]. Considering these findings, some authors advise against escalating maintenance steroid doses for AAV patients with concurrent SARS-CoV-2 infection, recommending a continuation of a gradual taper. Furthermore, there is advocacy for delayed re-dosing of rituximab during induction [17].

Despite these considerations, COVID-19 can potentially trigger a flare due to a hyperinflammatory state and may benefit from an escalation of steroid-based regimens [13]. After weighing the available evidence, given her stable renal function and low suspicion of DAH, we opted to continue the steroid taper for our patient. Her observed improvement aligns well with the above recommendation.

CMV DNAemia was subsequently discovered in our patient, with possible pneumonia contributing to the worsening respiratory status. Although the incidence varies widely, CMV has been estimated to cause 1%-11.7% of all major infectious episodes in patients treated for AAV [14,15]. Our patient’s advanced age, recent high steroid dose usage, renal impairment from vasculitis activity, low albumin, low total protein, elevated baseline CRP, and low hemoglobin levels placed her at high risk of CMV viremia and infection following induction therapy [16,17]. CMV viremia by itself has been associated with poor outcomes in patients with AAV, including increased length of ICU stay [18], emphasizing the significance of prompt diagnosis and treatment. 

A major challenge in diagnosing CMV in transplant patients, from which most of our knowledge stems, is distinguishing between asymptomatic viral shedding or antigenemia and clinically significant tissue invasion. This distinction determines whether intravenous ganciclovir or oral valganciclovir is indicated [19]. Additionally, CMV's ability to mimic other conditions, such as classical pneumonia and viral pneumonitis, adds to the diagnostic complexity. Although we were unable to definitively confirm tissue invasive disease in our patient, given the clinical setting and a high index of suspicion, we promptly initiated antiviral therapy. 

The next obstacle involves navigating immunosuppression while treating CMV infection. The traditional recommendation from transplant literature advises withdrawing or tapering immunosuppression [19]. However, applying similar conclusions to vasculitis patients may not be suitable, as the goals differ. In transplantation, the aim is to prevent inflammation, whereas in vasculitis, active suppression of inflammation is sought. 

Moreover, there has been a shift in understanding CMV reactivation, moving away from the previous notion of impaired immune responses to the emerging concept that it may occur during rapid immune recovery, similar to immune reconstitution inflammatory syndrome (IRIS). Initially observed in HIV patients, IRIS has been documented in various immunosuppressed states following abrupt withdrawal of immune suppression, including solid organ transplant patients, neutropenic individuals, and after discontinuation of TNF-α agonists [20,21]. It has been proposed for isolated autoimmune conditions such as drug-induced bullous autoimmune diseases and rheumatoid vasculitis, where CMV infections were seen following the withdrawal of steroids. Patients with low baseline CD4 T-cell counts and high HIV viral loads in HIV patients and lymphocyte counts and NLR at baseline for drug-induced bullous diseases have been noted to be predisposed to this phenomenon [22,23]. A similar mechanism may have played a role in our patient here, who had low lymphocyte counts at baseline. Her rapid steroid taper coupled with rituximab dosing delay with the treatment of COVID-19 may have caused rapid immune recovery, triggering the reactivation of CMV infection. This may explain why increasing the steroid dose while initiating antiviral treatment led to improvement in our patient. 

Given the rarity of AAV, it is challenging to gather high-quality evidence. Nevertheless, given its distinctive inflammatory nature, the importance of prospective multicenter trials cannot be overstated. These trials are crucial for investigating the role of IRIS in revealing opportunistic viral infections in future studies, particularly in understanding the impact of immunosuppression.

For maintenance therapy, the first-line option is rituximab. Second-line agents include azathioprine and methotrexate, with leflunomide and mycophenolate mofetil as the third line [24]. Given the multiple infectious complications with severe liver injury, our options for this patient are quite limited. Recent trials have shown promising results for avacopan, with non-inferior results to steroids in maintaining sustained remission, although the long-term safety profile is not well known [25]. There is also a conditional recommendation for the use of IVIG in patients who have not responded adequately to conventional immunosuppression with recurrent infections and hypogammaglobulinemia [24]. Following extensive multidisciplinary discussions, the potential use of avacopan and intravenous immunoglobulin was deliberated as additional treatment options. Currently, our approach involves vigilant monitoring for any signs of disease flare while concurrently sustaining steroid therapy. 

## Conclusions

In patients with AAV, immunosuppressive therapies such as rituximab and corticosteroids increase the risk of severe, potentially fatal COVID-19 infections and opportunistic viral infections like CMV. Therefore, maintaining a broad differential diagnosis is essential when evaluating hemoptysis in this population. Clinicians must remain vigilant, as viral infections can mimic various clinical conditions, complicating the diagnostic process.

While immunosuppression heightens infection risk, infections themselves may also precipitate vasculitis relapses. Additionally, rapid immune reconstitution can provoke pathogen-specific immune responses to latent or subclinical infections, akin to IRIS. Consequently, decisions regarding the withdrawal or intensification of immunosuppressive therapy must be carefully individualized based on the evolving clinical picture. Regular reassessment and dynamic adjustment of treatment strategies are critical for optimizing patient outcomes in these complex scenarios.
